# INEQUITY IN SCHOOL ATTENDANCE IN THE JIM CROW SOUTH: IMPLICATIONS FOR BLACK–WHITE DISPARITIES IN COGNITIVE FUNCTION

**DOI:** 10.1093/geroni/igad104.0442

**Published:** 2023-12-21

**Authors:** Katrina Walsemann, Stephanie Ureña, Mateo Farina, Jennifer Ailshire

**Affiliations:** University of Maryland, College Park, College Park, Maryland, United States; University of Maryland, College Park, Maryland, United States; University of Southern California, Los Angeles, California, United States; University of Southern California, Los Angeles, California, United States

## Abstract

Education’s contribution to Black–White disparities in cognitive function remains unclear due to data limitations that capture systemic educational inequities in the Jim Crow South. We include differential rates of school attendance across race, years, and states in the Jim Crow South. We linked state-level data on school attendance from the 1919 to 1954 Biennial Surveys of Education to the HRS. Sample restricted to Black and White older adults who attended school in the Jim Crow South in the study period (n = 4,343). We examined total cognitive function, episodic memory, and working memory. Total years of schooling explained 28%–33% of the Black–White disparity in all three outcomes. Differential rates of school attendance explained 41%–55% of the Black–White disparity in these outcomes. Our study highlights the importance of using a more refined measure of schooling for understanding the education–cognitive health relationship.

